# Increased risk of being diagnosed with endometriosis in patients with Systemic lupus erythematosus: a population-based cohort study in Taiwan

**DOI:** 10.1038/s41598-022-17343-4

**Published:** 2022-08-03

**Authors:** Yi-Hung Sun, Pui-Ying Leong, Jing-Yang Huang, James Cheng-Chung Wei

**Affiliations:** 1 Division of Gynecologic Oncology, Department of Obstetrics and Gynecology, Chimei Hospital, No. 901, Zhonghua Rd., Yongkang Dist., Tainan City, 710402 Taiwan; 2 Institute of Medicine, College of Medicine, Chung Shan Medical University, No. 110, Sec. 1, Jianguo N. Rd., South District, Taichung City, 40201 Taiwan; 3 Department of Allergy, Immunology and Rheumatology, Chung Shan Medical University Hospital, Taichung City, 40201 Taiwan; 4 Department of Medical Research, Chung Shan Medical University Hospital, No.110, Sec.1, Jianguo N. Rd., Taichung City, 40201 Taiwan; 5 Graduate Institute of Integrated Medicine, China Medical University, No. 110, Sec. 1, Jianguo N. Rd., South District, Taichung City, 40201 Taiwan

**Keywords:** Diseases, Medical research, Rheumatology

## Abstract

Epidemiological study shows inconsistent results in the association between endometriosis and Systemic lupus erythematosus (SLE). We conducted a nationwide retrospective cohort study and analyzed data from the Taiwan Longitudinal Health Insurance Research Database 2000 (n = 958,349) over a 13-year follow-up period (2000–2013). After matching 1930 SLE women with 7720 non-SLE women in a 1:4 ratio by age, we used Cox proportional hazard regression to calculate the adjusted hazard ratio (aHR) for endometriosis diagnosed after SLE. We also used a diagnosis of endometriosis with previous gynecologic surgery codes as secondary outcomes and performed sensitivity analyses using a landmark analysis. After adjustment for age, urbanization, income, length of hospital stay, and comorbidities in the age-matched group, women with SLE had a higher risk of endometriosis than women without SLE (aHR 1.32, 95% CI 1.02–1.70). When we defined endometriosis as patients with an ICD-9 endometriosis code after undergoing gynecologic surgery, the increased risk of endometriosis in patients with SLE was not significant. Our findings suggest that the risk of endometriosis was significantly elevated in the cohort of women with SLE compared with the age-matched general cohort of women. The burden of endometriosis in SLE patients requires special attention.

## Introduction

Systemic Lupus Erythematosus (SLE) is a chronic autoimmune disease with diverse clinical manifestations^1^. Gender, ethnicity, age of onset, and socioeconomic class strongly influence the occurrence of SLE, with large geographic differences in reported incidence and prevalence^2,3^. The contributions of genetic variation, immunity, the female hormone, and sex chromosomes have been extensively discussed in research^4^. There are also many studies exploring the association between SLE and female-specific disease.

Endometriosis is also a chronic multifactorial disease associated with systemic inflammatory response, affecting women of reproductive age and causing a huge clinical burden of chronic pelvic pain, abnormal uterine bleeding, and infertility^5,6^. Multiple articles suggest that female sex hormones may affect the maintenance of immunity or the development of autoimmune diseases^7,8^.

Epidemiological studies suggest a strong link between endometriosis and female-dominated autoimmune disease^9,10^. Several published studies have also analyzed the risk of SLE in women with endometriosis, but not all findings support a significant association between the two diseases^11–15^. According to a recent meta-analysis, only a few studies under review can provide high-quality evidence to support a significant association between endometriosis and SLE. Due to the small size of most of the included studies, the potential for spurious associations is high, and their limited statistical power also neutralizes important associations found in other well-designed large studies in meta-analyses. Furthermore, some of the included studies were cross-sectional and case–control studies, possibly biased by suboptimal control selection^16^. Therefore, we conducted a nationwide retrospective cohort study to assess the risk of endometriosis in women diagnosed with SLE by age matching methods and also adjusted by the degree of urbanization, status of income, and co-morbidities.

## Methods

### Study population

**Figure 1:**
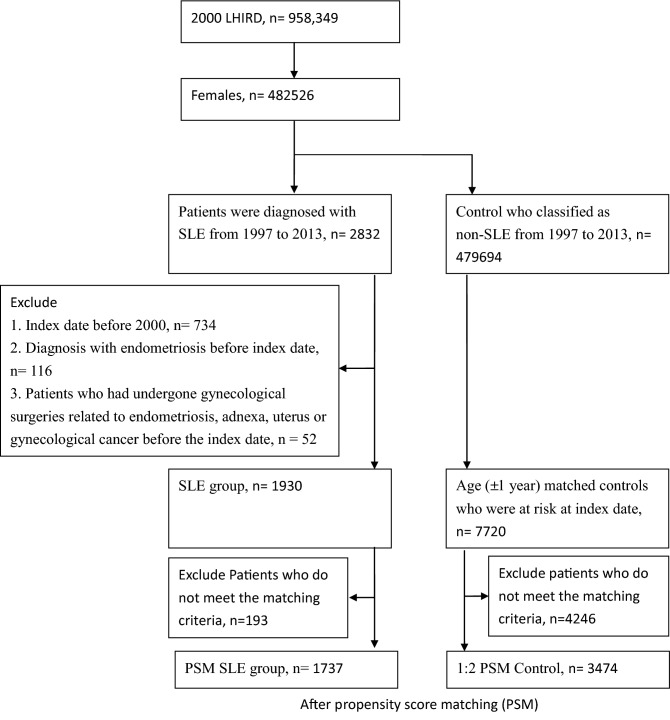
Flowchart of enrollment of the SLE cohort and the non-SLE cohort from the Longitudinal Health Insurance Database, 1997–2013.

This study had a retrospective cohort design (Fig. 1). The primary data were from the Taiwan National Health Insurance Research Database (NHIRD), which is a public database with de-identified participant data. The National Health Insurance (NHI) program in Taiwan has been launched in 1995. The 2000 longitudinal health insurance research database (2000 LHIRD) comprised 1,000,000 beneficiaries who were stratified sampled by age and sex from the population covered by the NHI system (23,753,407 individuals, 99% of populations in Taiwan) in the year 2000. Therefore, there were no differences in the percentages of gender and ages between NHIRD and LHIRD. In 2000 LHIRD, Physicians recorded diagnosis codes based on the International Classification of Diseases, 9th Revision, Clinical Modification (ICD-9-CM). Bureau of National Health Insurance validated all claims data to ensure accuracy. In this study, we used the 1997–2013 claim data in the 2000 LHIRD database without missing data. Because our research focused on gender-specific diseases, we only selected women for analysis. This study got an approval letter from the Institutional Review Board (IRB) of the Affiliated Chung Shan Medical University Hospital (CSMUH) before it started. They agreed to waive informed consent.

### Study cohort and identification of SLE

**Figure 2:**
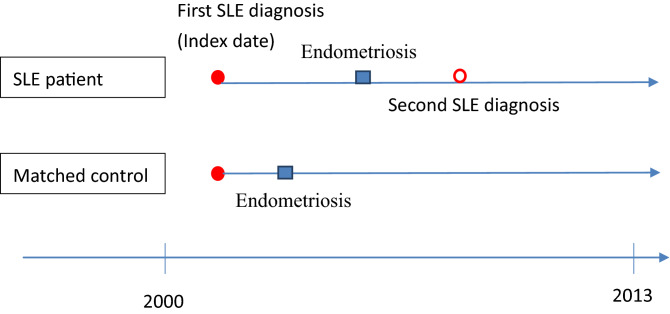
Diagram of follow-up from index date for SLE patients and matched comparators.

We identified a total of 2832 women with documented SLE diagnoses (ICD-9 code 710.0) between 1997 and 2013 as the SLE cohort and defined the index date as the first diagnosis of SLE. Representation of SLE patients by ICD-9 codes alone still lacks validation. To ensure an accurate diagnosis, the SLE cohort had to have an SLE diagnosis within one year of hospital admission or two or more outpatient visits. In this design, the secondary diagnosis of SLE was a clue to the accuracy of SLE diagnosis. From this aspect, the first diagnosis of SLE should be the index date (Fig. 2). In this study, observations were made between 1997 and 2013, and left-censored and truncated data may have biased estimates of causal effects^17,18^. To reduce this bias, we excluded prevalent SLE patients diagnosed with SLE before 2000 (n = 734) and patients with endometriosis (ICD-9: 617) diagnosed before the index date (n = 116). In addition, we wanted to rule out the effect of surgery on the female organs and the possibility that the patient already had endometriosis-related diseases or cancer. Therefore, we excluded patients who had undergone gynecological surgery related to endometriosis, adnexa, uterus, or gynecological cancer (Supplementary Table S1) before the index date (n = 52).

### Outcome and potential confounders

We defined the primary outcome as the diagnosis of endometriosis. The diagnostic code for endometriosis (ICD-9: 617) may be derived from clinical evidence or surgical intervention. According to the definition of endometriosis diagnosis, the surgery that allows direct visualization and pathology reporting is the gold standard for diagnosis. Because not every patient with underlying endometriosis is a candidate for surgical intervention, a diagnosis based on clinical evidence alone should not be underestimated. However, validation of the NHIRD database for endometriosis diagnoses is lacking. To strengthen the clinical evidence for endometriosis, we included only women with at least one documented hospital admission or two outpatient visits. Since the surgical diagnosis may lead to diagnostic delay and underestimation, we used surgical diagnosed endometriosis as a surrogate outcome for sensitivity analysis. We defined surgically diagnosed endometriosis as a patient with an endometriosis diagnostic code and a record of gynecologic or obstetric surgery related to the ovary or uterus. We tracked all individuals in the SLE and non-SLE groups from the index date until the event of endometriosis or the end of this study. We considered endometriosis diagnosed between the first and second SLE diagnosis to be a real event and do not exclude it to avoid unnecessary exclusion. Therefore, follow-up starts from the index date of these patients and matched comparators (Fig. 2). The medium follow-up time for the SLE group and age-matched non-SLE group was 92 and 88 months, respectively.

We defined factors that may be associated with endometriosis or SLE as potential confounders, including demographic variables (urbanization and low income), length of hospital stay, and comorbidities^2,6,7,16,23^. We identified the length of hospitalization and co-morbidities within one year before the index date and distinguished the degree of urbanization into urban, suburban, and rural^24^. We divided the length of hospital stays into four categories: 0 days, 1–6 days, 7–13 days, and more than 14 days. The co-morbidities we considered were hypertension, diabetes, hyperlipidemia, coronary artery disease, chronic liver diseases, chronic renal failure, cancer, vaginitis and vulvovaginitis, depression, and pelvic inflammatory disease^19–22^ (Supplementary Table S2).

### Comparison individuals

The non-SLE cohort included women who were not diagnosed with SLE between 1997 and 2013 (n = 479,715). We individually matched the non-SLE population with the SLE population in a ratio of 4:1 according to the age on the index date. Matched non-SLE patients and their matched SLE patients have the same index date and the same exclusion criteria. To balance baseline characteristics and minimize potential confounding bias, we performed propensity score matching (PSM) analysis via PROC PSMATCH in SAS software, selecting one SLE and two non-SLEs via a greedy nearest neighbor algorithm with 0.01 calipers. Propensity scores for SLE were estimated using logistic regression with predicted variables including age, urbanization, length of hospital stay, and comorbidities.

### Statistical analysis

We used absolute standardized difference (ASD) to present differences in socio-demographic characteristics (such as age, urbanization, and income), length of hospital stay, baseline comorbidities, and concomitant medication between the two groups. The concomitant medication we identified was between one year before and three months after the index date and included systemic corticosteroids, NSAIDs (excluding aspirin), aspirin, hydroxychloroquine, azathioprine, sulfasalazine Sulfapyridines, proton pump inhibitors (PPIs), histamine-2 (H2) inhibitors, and statins. When the ASD is less than 0.1^25^, we can regard the difference between the two groups as small. The incidence density rate and its 95% CI of endometriosis were calculated.

We also plotted the cumulative incidence curves of endometriosis between the two groups using the Kaplan–Meier method and determined differences in these curves by the log-rank test. We employed multivariate Cox proportional hazards regression models to assess adjusted HR (aHR) and crude HR (cHR) and associated 95% CIs of newly diagnosed endometriosis in the SLE group compared with the non-SLE group. Covariates employed in the multivariate model included age, urbanization, low income, length of hospital stay, and comorbidities. To assess possible study bias due to over-adjustment, we performed a multivariate Cox regression adjusted for baseline age, urbanization, and low income, and a multivariate Cox regression adjusted for baseline age, urbanization, low income, and comorbidities.

We performed a stratified analysis to assess statistical interactions^26^. Retrospective observational studies have limited us to finding new diagnoses of endometriosis in populations that are too young (right-censored) or old (left-censored or truncated). Therefore, we performed an age subgroup analysis to examine the incidence rate or risk of endometriosis. The aHR was calculated for specific age groups including < 20, 20–29, 30–44, and ≥ 45 years. In addition, we also stratified aHR by urbanization and co-morbidities to see if the association of SLE with endometriosis was at particular risk in specific populations.

Given the limitations of the retrospective design, we hoped to assess the data quality and correctness of diagnostic codes and reduce erroneous findings due to misclassification. Therefore, we performed a sensitivity analysis to see if the results would change significantly under different definitions. In terms of endometriosis, the diagnostic credibility of endometriosis is more acceptable for women undergoing surgical intervention. Hence, we defined patients with a diagnosis of endometriosis after gynecologic surgery as a secondary outcome and assessed adjusted hazard ratios for incidence in the study and control populations. We performed a landmark analysis and set a landmark time point at 24 months to explore the potential time-varying effects of SLE on endometriosis. We assessed the aHR and 95% CI for newly diagnosed endometriosis between index date and landmark time point and after the landmark time point in the SLE and non-SLE groups, respectively^27^. All statistical analyses were performed using SAS Version 9.4 (SAS Institute, Carey, North Carolina, USA). All methods are performed following relevant guidelines and regulations and confirmed in the STROBE checklist (Supplementary Table S3).

### Ethical approval

This study got an approval letter from the Institutional Review Board (IRB) of the Affiliated Chung Shan Medical University Hospital (CSMUH) before it started. They agreed to waive informed consent.

## Results

This study consisted of 1930 SLE patients and 7720 non-SLE patients in the 1:4 matched groups. After PSM, patients in the SLE group (n = 193) and the non-SLE group (n = 4246) who did not meet the matching criteria were excluded. Ultimately, there were 1732 SLE patients and 3474 non-SLE patients in the 1:2 PSM group. Table 1 shows the baseline characteristics among study groups. There was no significant difference in age, urbanization, low-income status, and some co-morbidities including hypertension, diabetes, and hyperlipidemia between control and SLE groups, regardless of before and after PSM (ASD < 0.1). In the 1:4 matched groups, patients with SLE had a higher proportion of hospitalization records and co-morbidities including coronary artery disease, chronic liver disease, chronic renal failure, cancer, vaginitis, vulvovaginitis, and depression compared with non-SLE patients (ASD > 0.1). In both of 1:4 matching and 1:2 PSM groups, patients with SLE had a higher proportion of usage of co-medication including systemic corticosteroids, NSAID, aspirin, hydroxychloroquine, azathioprine, sulfasalazine, PPI, and H-2 inhibitor (ASD > 0.1).

**Table 1:** Baseline characteristics among study groups.

	Before propensity score matching			After propensity score matching		
	Non-SLE n = 7720	SLE n = 1930	ASD	Non-SLE n = 3474	SLE n = 1737	ASD
**Age**			0.000			0.032
< 20	1001 (12.97%)	253 (13.11%)		451 (12.98%)	241 (13.87%)	
20–29	1657 (21.46%)	414 (21.45%)		776 (22.34%)	379 (21.82%)	
30–44	2195 (28.43%)	547 (28.34%)		993 (28.58%)	502 (28.90%)	
≥ 45	2867 (37.14%)	716 (37.10%)		1254 (36.10%)	615 (35.41%)	
**Urbanization**			0.070			0.000
Urban	4695 (60.82%)	1217 (63.06%)		2172 (62.52%)	1087 (62.58%)	
Sub-urban	2295 (29.73%)	528 (27.36%)		975 (28.07%)	479 (27.58%)	
Rural	730 (9.46%)	185 (9.59%)		327 (9.41%)	171 (9.84%)	
Low income	35 (0.45%)	14 (0.73%)	0.036	18 (0.52%)	10 (0.58%)	0.008
**Length of hospital stays**^a^			0.389			0.064
0 day	7144 (92.54%)	1566 (81.14%)		3078 (88.60%)	1524 (87.74%)	
1–6 days	397 (5.14%)	182 (9.43%)		321 (9.24%)	156 (8.98%)	
≥ 7 days	179 (2.32%)	182 (9.43%)		75 (2.16%)	57 (3.28%)	
**Co-morbidity**^b^						
Hypertension	885 (11.46%)	258 (13.37%)	0.058	399 (11.49%)	204 (11.74%)	0.008
Diabetes	470 (6.09%)	110 (5.70%)	0.017	198 (5.70%)	95 (5.47%)	0.010
Hyperlipidemia	523 (6.77%)	165 (8.55%)	0.067	268 (7.71%)	127 (7.31%)	0.015
Coronary artery disease	284 (3.68%)	122 (6.32%)	0.121	163 (4.69%)	84 (4.84%)	0.007
Chronic liver diseases	450 (5.83%)	269 (13.94%)	0.274	323 (9.30%)	164 (9.44%)	0.005
Chronic renal failure	49 (0.63%)	46 (2.38%)	0.144	20 (0.58%)	13 (0.75%)	0.021
Cancer	124 (1.61%)	68 (3.52%)	0.122	73 (2.10%)	43 (2.48%)	0.025
Vaginitis and vulvovaginitis	621 (8.04%)	226 (11.71%)	0.123	400 (11.51%)	190 (10.94%)	0.018
Depression	569 (7.37%)	298 (15.44%)	0.256	397 (11.43%)	204 (11.74%)	0.010
Pelvic inflammatory disease	394 (5.10%)	135 (6.99%)	0.079	255 (7.34%)	114 (6.56%)	0.031
**Co-medication**^b^						
Systemic corticosteroids	1915 (24.81%)	1394 (72.23%)	1.078	912 (26.25%)	1226 (70.58%)	0.990
NSAIDs (exclude Aspirin)	5204 (67.41%)	1705 (88.34%)	0.521	2453 (70.61%)	1527 (87.91%)	0.437
Aspirin	729 (9.44%)	435 (22.54%)	0.363	364 (10.48%)	353 (20.32%)	0.275
Hydroxychloroquine	23 (0.30%)	888 (46.01%)	1.289	12 (0.35%)	794 (45.71%)	1.279
Azathioprine	3 (0.04%)	178 (9.22%)	0.448	2 (0.06%)	147 (8.46%)	0.426
Sulfasalazine	13 (0.17%)	109 (5.65%)	0.331	7 (0.20%)	99 (5.70%)	0.329
PPI	210 (2.72%)	182 (9.43%)	0.284	115 (3.31%)	130 (7.48%)	0.186
H2 inhibitor	1599 (20.71%)	645 (33.42%)	0.289	760 (21.88%)	553 (31.84%)	0.226
Statin	299 (3.87%)	117 (6.06%)	0.101	145 (4.17%)	83 (4.78%)	0.029

^a^The length of hospital stays, comorbidities were identified within 1 year before the index date.

^b^The co-medication was identified between one year before and three months after the index date.

**Figure 3:**
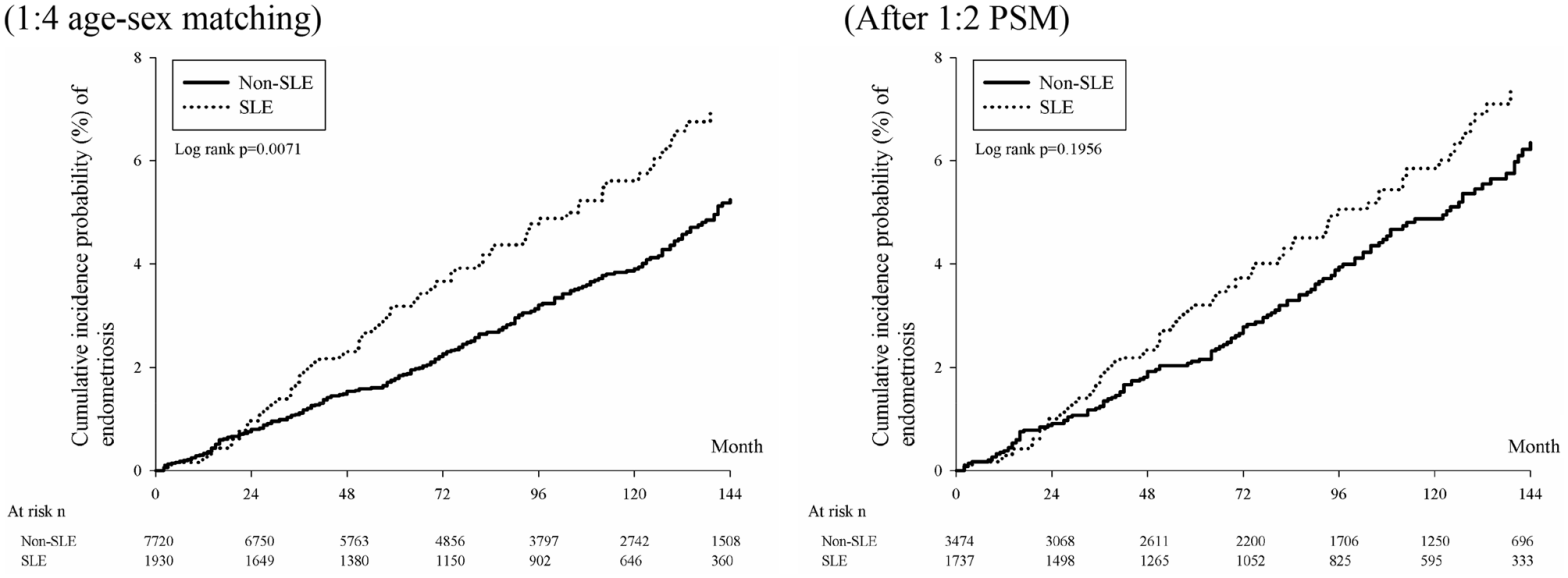
The cumulative incidence curves of endometriosis were plotted by the Kaplan–Meier method.

Table 2 shows the incidence rate (per 10,000 person-months) of endometriosis before PSM was 4.92 (95% CI 3.97–6.11) in the SLE group and 3.51 (95% CI 3.10–3.98) in the non-SLE group, respectively. The crude relative risk was 1.41 (95% CI 1.10–1.80) in the SLE group compared to the non-SLE group before PSM. After PSM, the crude relative risk was 1.20 (95% CI 0.91–1.58) and was not significant. In the multiple-variables regression adjusted with age, urbanization, and low income, the adjusted HR was 1.41 (95% CI 1.10–1.81). The patients with SLE had a 1.32 (95% CI 1.02–1.70) times higher risk of endometriosis after adjusting for sex, age, urbanization, length of hospitalization, and co-morbidities than the individuals without SLE before propensity score matching. The relative risk of endometriosis was 1.20 (95% CI 0.91–1.58) in patients with SLE compared with the propensity score-matched non-SLE individuals. The cumulative incidence curves of endometriosis were plotted by the Kaplan–Meier method and the SLE cohort showed a higher cumulative incidence of endometriosis than that of the non-SLE cohort in the 1:4 age matching group (Fig. 3).

**Table 2:** Incidence of endometriosis in study groups.

	Before propensity score matching		After propensity score matching	
	Non-SLE n = 7720	SLE n = 1930	Non-SLE n = 3474	SLE n = 1737
Follow up person months	701,176	168,567	317,762	153,973
New endometriosis case	246	83	136	79
Incidence rate^a^ (95% CI)	3.51 (3.10–3.98)	4.92 (3.97–6.11)	4.28 (3.62–5.06)	5.13 (4.12–6.40)
Crude relative risk (95% CI)	Reference	1.41 (1.10–1.80)	Reference	1.20 (0.91–1.58)
Adjusted hazard ratio^b^ (95% CI)	Reference	1.41 (1.10–1.81)		
Adjusted hazard ratio^c^ (95% CI)	Reference	1.32 (1.02–1.70)		

^a^Incidence rate, per 10,000 person-months.

^b^Multivariate Cox regression adjusted for baseline age, urbanization, and low income.

^c^Multivariate Cox regression adjusted for baseline age, urbanization, low income, and comorbidities.

We also conducted a subgroup analysis stratified by age, degree of urbanization, and co-morbidities to clarify the interactions and effects of SLE on endometriosis in each subgroup (Table 3). The result showed that patients with SLE had significant higher risk of endometriosis in the sub-group including age between 30 and 44 years (aHR 1.46, 95% CI 1.03–2.08), lived in rural areas (aHR 3.25, 95% CI 1.51–7.00), without diabetes (aHR 1.32, 95% CI 1.02–1.71), without hyperlipidemia (aHR 1.33, 95% CI 1.02–1.73), without coronary artery diseases (aHR 1.33, 95% CI 1.02–1.72), without chronic liver diseases (aHR 1.35, 95% CI 1.03–1.77), without chronic renal failure (aHR 1.32, 95% CI 1.02–1.70), without cancer (aHR 1.30, 95% CI 1.00–1.69) and without depression (aHR 1.44, 95% CI 1.10–1.90). However, we did not find a significant interaction between SLE exposure and other factors on endometriosis risk.

**Table 3:** Stratified analysis of endometriosis risk between SLE and non-SLE groups before propensity score matching.

Sub-group	Incidence rate^a^ (95% CI) of endometriosis		aHR^b^ (95% CI)
	Non-SLE (n = 7720)	SLE (n = 1930)	
**Age**			p for interaction = 0.2433
< 20	1.67 (1.04–2.68)	2.84 (1.36–5.97)	1.78 (0.70–4.52)
20–29	4.66 (3.69–5.88)	5.03 (3.21–7.89)	0.98 (0.58–1.67)
30–44	5.96 (4.98–7.13)	10.15 (7.65–13.47)	1.46 (1.03–2.08)
≥ 45	1.58 (1.15–2.16)	1.53 (0.79–2.94)	1.09 (0.52–2.29)
**Urbanization**			p for interaction = 0.1048
Urban	3.76 (3.22–4.38)	4.63 (3.51–6.10)	1.14 (0.82–1.59)
Sub-urban	3.21 (2.52–4.09)	4.63 (3.02–7.10)	1.42 (0.86–2.37)
Rural	2.80 (1.76–4.44)	7.93 (4.50–13.96)	3.25 (1.51–7.00)
**Co-morbidity**			
**Hypertension**			p for interaction = 0.7905
Without	3.77 (3.32–4.28)	5.26 (4.22–6.56)	1.29 (0.99–1.68)
With	1.15 (0.58–2.30)	2.17 (0.82–5.79)	1.89 (0.44–8.12)
**Diabetes**			p for interaction = 0.9807
Without	3.64 (3.21–4.13)	5.07 (4.07–6.30)	1.32 (1.02–1.71)
With	1.09 (0.41–2.90)	2.31 (0.58–9.24)	1.20 (0.14–10.42)
**Hyperlipidemia**			p for interaction = 0.8225
Without	3.64 (3.21–4.13)	5.08 (4.07–6.33)	1.33 (1.02–1.73)
With	1.27 (0.53–3.04)	3.08 (1.16–8.20)	0.88 (0.19–4.07)
**Coronary artery disease**			p for interaction = 0.7046
Without	3.56 (3.14–4.04)	5.03 (4.04–6.27)	1.33 (1.02–1.72)
With	2.09 (0.87–5.01)	3.11 (1.00–9.66)	0.89 (0.14–5.66)
**Chronic liver diseases**			p for interaction = 0.4346
Without	3.50 (3.07–3.98)	5.05 (4.02–6.34)	1.35 (1.03–1.77)
With	3.69 (2.23–6.13)	4.09 (2.13–7.87)	0.98 (0.42–2.29)
**Chronic renal failure**			p for interaction =
Without	3.52 (3.11–3.99)	5.02 (4.04–6.22)	1.317 (1.018–1.704)
With	No endometriosis case	No endometriosis case	
**Cancer**			p for interaction = 0.5759
Without	3.52 (3.11–3.99)	4.89 (3.93–6.09)	1.30 (1.00–1.69)
With	2.30 (0.58–9.20)	5.91 (1.91–18.32)	9.9 (0.69–142.46)
**Vaginitis and vulvovaginitis**			p for interaction = 0.9306
Without	3.39 (2.97–3.87)	4.74 (3.75–5.99)	1.31 (0.99–1.74)
With	4.77 (3.30–6.92)	6.25 (3.63–10.76)	1.27 (0.64–2.55)
**Depression**			p for interaction = 0.1678
Without	3.35 (2.93–3.82)	4.79 (3.78–6.06)	1.44 (1.10–1.90)
With	5.69 (3.90–8.30)	5.73 (3.39–9.68)	0.79 (0.40–1.57)
**Pelvic inflammatory disease**			p for interaction = 0.9083
Without	3.34 (2.93–3.81)	4.65 (3.69–5.85)	1.32 (1.00–1.73)
With	6.33 (4.28–9.37)	8.08 (4.47–14.58)	1.47 (0.70–3.09)

^a^Per 10,000 person-months.

^b^Adjusted for baseline demographic variables, length of hospital stay, and comorbidities.

Table 4 represents the sensitivity analysis and the landmark analysis. It shows that the aHR in the first 24 months was non-significant 1.17 (95% CI 0.67–2.04), but 24 months after the first diagnosis of SLE, the aHR was modified to significant 1.45 (95% CI 1.08–1.95). However, the P for time-varying is not significant (time-varying p = 0.4224). When we defined endometriosis as a patient with an ICD-9 code of endometriosis and undergoing gynecological surgery, the increased risk of endometriosis in patients with or without SLE is not significant.

**Table 4:** Sensitivity analysis of endometriosis risk in SLE and non-SLE patients.

	Before propensity score matching			After propensity score matching		
	Incidence rate^a^ (95% CI)		aHR	Incidence rate^a^ (95% CI)		aHR
	Control	SLE		Control	SLE	
Primary outcome: ICD-9 code	3.51 (3.10–3.98)	4.92 (3.97–6.11)	1.32 (1.02–1.70)	4.28 (3.62–5.06)	5.13 (4.12–6.40)	1.20 (0.91–1.59)
**Landmark analysis**						
0–24 months	3.47 (2.69–4.49)	4.14 (2.58–6.66)	1.17 (0.67–2.04)	3.98 (2.78–5.69)	4.30 (2.64–7.03)	1.11 (0.60–2.04)
≥ 24 months	3.56 (3.07–4.13)	5.60 (4.39–7.14)	1.451 (1.08–1.95)	4.40 (3.61–5.37)	5.84 (4.55–7.49)	1.32 (0.96–1.82)
p for time varying			0.4224			0.5984
**Alternative outcome**						
ICD-9 code with surgery for gynecological disease	1.81 (1.52–2.15)	2.33 (1.71–3.18)	1.24 (0.86–1.79)	2.21 (1.75–2.79)	2.49 (1.82–3.41)	1.15 (0.77–1.69)

*aHR* adjusted hazard ratio, controlling age, urbanization, hospital stay, and comorbidity at baseline.

^a^Incidence rate, per 10,000 person-months.

## Discussion

This nationwide retrospective cohort study revealed a statistically significant association between SLE and endometriosis. After adjusting for age, urbanization, low-income status, length of hospitalization, and co-morbidities, the aHR of endometriosis for SLE patients was 1.32 (95% CI 1.02–1.70). However, after further PSM, the aHR of endometriosis for SLE patients was not significant (1.20, 95% CI 0.91–1.59). In addition, when we limit the diagnosis of endometriosis to patients who have undergone surgery, its correlation with SLE is not significant.

According to the meta-analysis reported by Shigesi et.al, the major problem of current epidemiological studies regarding the association between endometriosis and autoimmune diseases is the limited case number. Due to limited statistical power, this may lead to a non-significant result. Other issues are suboptimal control selection and the difficulty in determining the order of disease progression and manifestation^16^. In our study, we used the cohort study design and PSM to analyze the risk of endometriosis. After analyzing the baseline characteristics between the study groups (Table 1), we can conclude that patients with SLE have a significantly higher rate of co-morbidities and medication usage in the age-matched group before PSM. The PSM can minimize the potential confounding bias by measuring co-variates. However, overmatching could also leads to biased conclusions. The procedure of PSM excluded the patients with extreme propensity scores might decrease the sample size and statistic power, and might increase the homogeneity between groups. From this perspective, age-matched non-SLE individuals were more likely general individuals. Based on the result before propensity score matching, the risk of endometriosis was significantly elevated in the cohort of women with SLE compared with the age-matched general cohort of women. After PSM, the sample size was reduced and a control group that was different from the general population was also selected.

Because of the limitation of non-invasive diagnostic tools for endometriosis, it is impossible to know exactly how many patients have endometriosis. Vice versa, the diagnostic code for endometriosis based on clinical symptoms and physical examination may also include patients who do not have endometriosis. Since surgical exploration is the standard diagnostic method for endometriosis, from the perspective of epidemiological research, it is more convincing to use the surgical code of endometriosis-related diseases to identify patients with endometriosis^16^. However, not all patients diagnosed with endometriosis require surgery. Therefore, patients with endometriosis who have never undergone surgery will be omitted, leading to underpowered results. The aHR (1.24, 0.86–1.79) of SLE patients with endometriosis and gynecological surgery is not significant, which may also be due to the small sample size and statistical power.

Endometriosis and SLE are both diseases that occur months to years before clinical diagnosis. According to our study, SLE patients have a higher incidence of being diagnosed with endometriosis in the future. The Kaplan–Meier curve in Fig. 2 shows the crude relative risk of endometriosis in SLE patients in the age-matched group is significantly increased when compared with non-SLE patients. Landmark analysis in Table 4 also shows that after 24 months from the index date, the incidence of endometriosis represented an upward trend. The association of SLE with endometriosis did not change significantly over time, as the time-varying P-value did not show significance. However, such results should also remind us that there is a significant delay in the diagnosis of endometriosis. The burden of endometriosis in SLE patients still requires special attention.

Table 4 also shows that SLE patients between the ages of 30 and 44 had a higher incidence of endometriosis compared with non-SLE patients. An epidemiological study in northeastern Italy showed a relatively high incidence of endometriosis and adenomyosis between the ages of 30 and 50^24^, which was comparable to our results. However, we did not find a significant moderating effect of age on the association between SLE and endometriosis (the p-value for interaction was not significant).

One of the limitations of our study is the possible misclassification of cases in the study cohort, as well as data validation. Further study about data verification for the diagnosis of SLE and endometriosis from NHIRD is still required. When dealing with this problem, we screen patients according to the actual situation of medical treatment. For hospitalized patients, patient-related diagnoses will be withheld due to the team review mechanism. For outpatients, more than one visit can indirectly illustrate the need for medical attention and the authenticity of the diagnosis. Another question is whether one diagnosis affects another. Immunosuppressed women with SLE may not be able to undergo extensive surgery or may not need surgery due to the use of medication to improve symptoms. Therefore, we can hypothesize that when an endometriosis diagnosis requires surgery, these patients may be underestimated as ineligible for surgery due to other medical problems. In addition, as the population of this study is only Taiwan residents, our results might only apply to East Asia populations if SLE varies by race or ethnicity. The lack of information about birth history, menopausal status, or history of hormone therapy can also affect and confound our findings. Another limitation is that SLE patients have a higher rate of medication usage and those medications are specific to SLE patients and cannot be adjusted. Because data from healthcare databases were not from randomized trials, drug use may be associated with differences in disease severity. Therefore, it is difficult for this study to assess the actual mediating effect of the drug. The treatment for SLE also varies according to the severity and clinical manifestations of the disease. We still need another study to assess the effect of drugs on these two diseases and to find out whether different drugs in SLE patients affect the development of endometriosis.

The advantages of our research include a large population-based cohort study and 13 years of follow-up, effective diagnostic codes for endometriosis, and methods for defining SLE that can reduce the possibility of misclassification and underestimation, as well as time assumption. The 1:4 age matching design and patients selection methods we used are suitable for adjusting confounding factors without loss of representativeness of the general population. Taken together, the framework we used in this study helped us to obtain a sufficient number of cases and reliable confidence intervals.

## Conclusion

In conclusion, the risk of endometriosis was significantly higher in SLE patients than in general populations. Further prospective studies of patients newly diagnosed with SLE, including long-term follow-up and careful gynecological evaluations, are important for clarification.

## Supplementary Information

Supplementary Tables.

## Data Availability

The data underlying this article are available in *Taiwan Longitudinal Health Insurance Research Database*. The datasets were derived from sources in the public domain: https://nhird.nhri.org.tw/apply_00.html.
